# Supplementary material for: Waste By-Product of Grape Seed Oil Production: Chemical Characterization for Use as a Food and Feed Supplement
Source: Life (Basel). 2023 Jan 24;13(2):326. doi: 10.3390/life13020326 (PMC9958947; doi:10.3390/life13020326)
Supplement: Supplementary file 1 [file life-13-00326-s001.zip › life-2127985-supplementary.pdf]

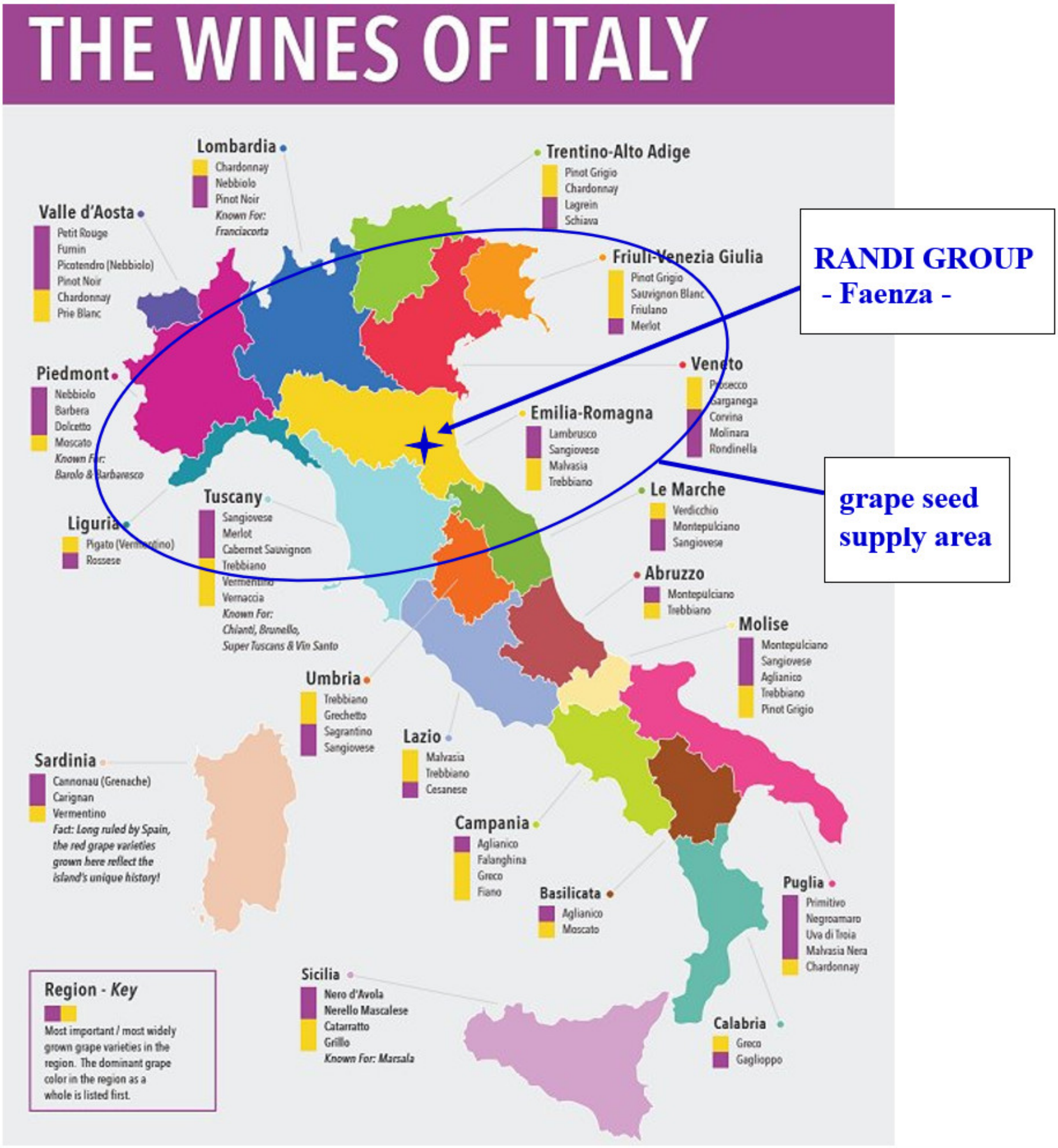

Figure S1. The wines of Italy: Randi Group supply area.

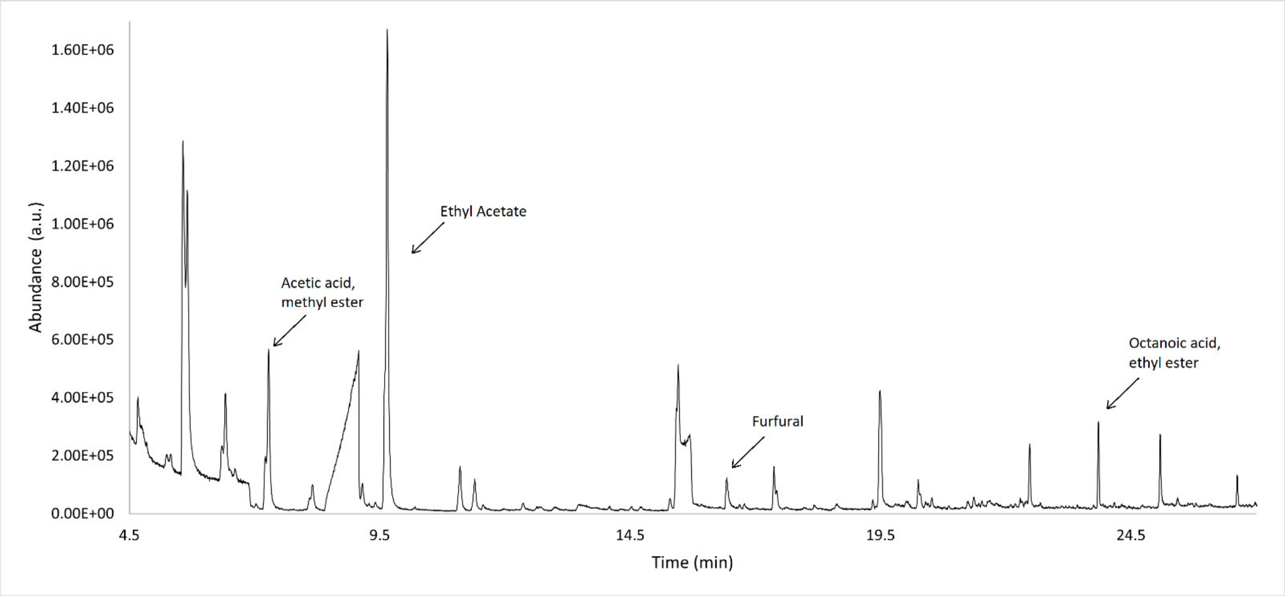

Figure S2. Total ion current chromatogram of VOCs from the DGS sample, obtained using HS-SPME-GC-MS.
